# Some Remarks on the Ætiology, Pathology and Treatment of Malarial Hæmatinuria

**Published:** 1894-07

**Authors:** T. F. Oates

**Affiliations:** Mexia, Texas


For Texas Medical Journal.

### SOME REMARKS ON THE ÆTIOLOGY, PATHOLOGY AND TREATMENT OF MALARIAL HÆMATINURIA.

T. F. OATES, M. D., MEXIA, TEXAS.

THE scarcity of literature upon malarial hæmatinuria is without a parallel in the history of medicine; why this scarcity, is a problem difficult of solution. It can not be the infrequency of its occurrence or the circumscribed habitat of the disease; for it occurs annually, and perhaps in almost all the Southern and Western States, as well as Africa, South America and other southern countries. Indeed, its habitat must be coextensive with that of malarial fevers. Neither can it be the mildness of the malady, for its fatality makes it a dreaded monster wherever it is known. Epidemics of great virulence are not infrequent in the bottom country and swampy districts of the South. Whatever be the cause, this scarcity is quite apparent. Now and then an article in some medical journal by a country doctor is published, and this is the main source and almost the limit of the present knowledge on the subject. So far as I know there is not a single exhaustive scientific treatise upon the ætiology, symptomatology and treatment of malarial hæmatinuria.

Therefore, in this paper, should I go far astray, I would not go counter to any great scientific deliverance upon the subject. Our medical lecturers and writers are on record about as follows: Malarial hæmatinuria is a malarial disease, therefore, treat with calomel and quinine. And a majority of the profession who are neither lecturers nor writers, were they to express themselves would drawl out in parrot-like style the same sentence. Upon the ætiology of black jaundice, as it is vulgarly termed, perhaps but little difference of opinion obtains. It is not positively settled that malaria is even the primary cause. No proof to that effect has been brought forward, yet it is, without formal proof, the universally accepted opinion that the germ of intermittent and remittent fevers is also the primary cause of this disease. Further than this there is perhaps no general agreement as to the ætiology of malarial hæmatinuria. By the majority of medical men there is no other than the primary cause, no other cause than the micro-organisms of malarial intermittent and remittent fevers. I am in accord with the prevalent opinion in regard to the original cause, but the immediate causative agency is quite an obscure and complex condition, more comprehensive than the simple microbe. The bacterium or hæmatozoon may be a factor of the secondary cause.

It is only a factor. It does not comprise the whole of that cause. Malarial hæmatinuria is the sequel of malarial intermittent or remittent fever and not one of those fevers. Pathologically it is like the devastated condition of a country through which a great army has been marching and foraging, leaving here and there straggling soldiers, who continue pilfering, annoying and exhausting the already impoverished country.

There is a long chain of pathological processes, the first link of which is that of intermittent or remittent fever, the last of which is hæmatinuria, and the links of this chain, one after another, are forged something like the following: At first the blood is impregnated, the entire system is permeated with malarial hæmatozoa. Sporulation takes place, and a chill ensues; quinine is administered, and the offending germs are dead. The nerve shock as manifested by the febrile action passes off, and the first link is wrought.

The spores, no doubt, have been robbed of vital germinal force by the antiperiodic. An unhealthy second generation of plasmodian life has begun. Unhealthy and few in number, this second generation gives rise to no appreciable disturbance in the economy of the patient, and so it continues to the third generation on or about the seventh day; or if the spores are affected, more decidedly devitalized till a very few of them generate into a yet more sickly life, it may require six generations or fourteen days. This we often see.

But the third or sixth generation, as the case may be, is a large army of stalwart fellows, and they produce a repetition of intermittent fever, or perhaps if more numerous and yet more robust, the severity of pernicious or congestive chill may be observed. But again quinine is administered, and the malarial parasite is crippled, and perhaps for three or six generations, as before. Thus it continues for months, usually from early summer to early autumn, and while the number of germs in the blood is thus kept down to a small insignificant number, yet that number is living on the blood tissue, devitalizing and poisoning it with their leucomains or toxines. The blood finally becomes deficient in oxygen and surcharged with carbon dioxide. Impending dissolution of the vital fluid is apparent; in fact, dissolution of the red corpuscles has begun, and we have the disease known as malarial hæmatinuria, manifested by the port-wine urine, canary skin, buccal mucous membrane, and conjunctiva; by nausea, fever and constipated bowel, and this is the last link in the chain of pathological process. Thus we have not so much a germ to treat, but a pathological condition consequent upon the long continued habitation and procreation of germ life in the economy. The blood has been robbed day by day, little by little, of its oxygen carrying power, and we have, as already has been said, a deficiency of oxygen in the blood, a proportionately increased quantity of carbon dioxide, disintegration of red blood cells, red urine from excretion of those broken down cells, which in the act of excretion sometimes become so abundant that a damming, or blocking up takes place in the kidneys, and we have anuria. The lining membrane of the bowel, and sometimes the stomach, is plastered over with these same disintegrated blood cells in the act of excretion by this tract. The mouth of bile duct is blocked up by the same, and we have absorption of bile into the blood. This absorption, together with the coloring matter from broken down cells, permeating the entire system, produces the yellow skin, conjunctiva, etc. We have, to be sure, a few malarial hæmatozoa. To treat black jaundice, therefore, is to correct the pathological condition just outlined. In this, as in many other conditions, we cannot treat the primary cause. The immediate cause demands our first attention. We have said the blood is deficient in oxygen. As to whether this deficiency of oxygen results directly from reduction of the oxygen carrying power by the absence of one of the essential constituents of the blood, or from reduction of carbonic acid carrying power, we shall not attempt to explain at length. The probability is that the deficiency of oxygen is caused by the failure of the blood to carry off the carbon dioxide, which in turn displaces the oxygen. What is that element that gives the blood tissue its solvent power over carbon dioxide, and thus secondarily over oxygen? The blood is a heterogeneous mixture of elements or compounds, and to retain its power to perform its full physiological function, each component part, element, or proximate principle must vary only within certain limits. When any one of these is increased or diminished beyonnd these limits, a diseased condition is the result. In the disease, or pathological condition now under consideration, the blood has been reduced in its alkaline phosphates to the extent that it no longer carries sufficient oxygen, and it is by virtue of the alkaline phosphates that the blood is especially a great oxygen carrier, whether it be directly or indirectly.

As to whether the dissolution of the red cells takes place by reason of the disturbed proportion of the component parts, caused by the diminished phosphates, or by reason of imperfect oxygenation consequent upon diminished phosphates, I shall not stop to inquire. It is sufficient to say that the immediate or proximate cause of the pathological condition present in malarial hæmatinuria is a diminished proportion of the alkaline phosphates. The pathology of this affection at once suggests the treatment; clearly, the first indication is to furnish the blood with abundance of the phosphates. But to do this effectually the alimentary tract must be swept of the unhealthy debris with which it is filled, then the phosphates may be administered. Synchronous with this the kidneys should be cleared out with a non-imitating diuretic.

Next our attention is addressed to that half-developed but well acclimated brood of malarial hæmatozoa; for this gives an anti-malarial. Excite nutrition with stomach and blood tonics, and furnish to the system easily appropriated nutrients. As representative of the alkaline phosphates I give sodium phosphate; as a non-irritating diuretic, acetate of potassium; Fowler's solution as the anti-malarial specific, and as a cathartic mercuric chloride.

Called to a case, diagnosis clear, I begin treatment by giving to an adult twenty-five or thirty grains of mercuric chloride; at the same time begin with fifteen grain-doses of potassium acetate, and twenty to thirty-grain doses of sodium phosphate at intervals of three hours; also half glass of sweet milk, containing a tablespoonful of brandy, at same intervals. The next day direct the same continued, except the mercuric chloride, which already will have accomplished its purpose, and give in addition Fowler's solution, four or five drops, in a half glass of milk punch, every three hours. I make no further change till the third or fourth day, when I usually give a prescription of tincture of chloride of iron and chlorate of potassium. I can not give the exact specific indications for the last prescription, but, in a general way, may say that it furnishes ingredients deficient in normal quantity. This plan of treatment has enabled me to report about fifteen consecutive cases without a single death. Some of this nnmber were mild, others of the severest type. I wish to call especial attention to the fact, which already I have attempted to emphasize, that the main indication in treatment is the administration of sodium phosphate freely and persistently from the beginning, for that meets the main pathological condition, namely: deficient oxygenation of the blood. I wish to call attention, also, to the fact that there is no great urgency for the administration of any anti-malarial, or rather any remedy that will kill malarial germs. But especially do I wish to express, in raised letters, my condemnation of the use of quinine, not that it fails to have its usual specific effect upon the micro-organisms, by any means. In the first place, these little blood animals are not so numerous as to create any great immediate alarm. In the second place, the blood tissue is devitalized to that extent that disintegration is rapidly going on—complete dissolution impending. Quinine is a protoplasmic poison. It inhibits functional activity of the blood. The blood is not in a condition to bear any drafts upon its physiological resources. It may kill the remaining micro-organisms, but at the same time in severe cases it will prove to be the "feather that breaks the camel's back." It may give the finishing touch to the devitalizing process to which the blood has been subjected so long.

For the well-acclimated germ of chronic malaria, arsenic is the ideal specific. It is not a protoplasmic poison; it does not inhibit functional activity of the blood; it favors constructive metamorphosis, and withal, as has been said, destroys the micro-organism of chronic malarial toxæmia.

---

For Texas Medical Journal.

