# Errata

**Published:** 1894-09

**Authors:** 


**Errata.**—In Dr. Oates' excellent article on "Malarial Hæmatinuria" in our July number, for "mercuric chloride" read "mercurus chloride." The error occurs in three places.

